# Supplementary material for: Genomic Characterization of a Plasmid-Free and Highly Drug-Resistant Salmonella enterica Serovar Indiana Isolate in China
Source: Vet Sci. 2024 Jan 20;11(1):46. doi: 10.3390/vetsci11010046 (PMC10819017; doi:10.3390/vetsci11010046)
Supplement: Supplementary file 1 [file vetsci-11-00046-s001.zip › vetsci-2766385-supplementary.pdf]

Supplementary files.

	Description	Scientific Name	Max Score	Total Score	Query Cover	E value	Per. Ident	Acc. Len	Accession
☑	Salmonella enterica subsp. enterica serovar Indiana strain S1467 chromosome, complete genome	Salmonella enterica s...	94721	1.885e+05	100%	0.0	100.00%	4998300	CP121189.1
☑	Salmonella enterica subsp. enterica serovar Indiana strain YZ20MCS6 chromosome, complete genome	Salmonella enterica s...	76269	1.789e+05	98%	0.0	99.84%	4990287	CP098829.1
☑	Escherichia coli strain EBJ003 chromosome, complete genome	Escherichia coli	71230	1.441e+05	98%	0.0	99.20%	5279212	CP086334.1
☑	Escherichia coli strain EC5 chromosome, complete genome	Escherichia coli	70969	1.313e+05	99%	0.0	99.08%	4801130	CP060969.1
☑	Escherichia coli O157:H16 strain Santai, complete genome	Escherichia coli O157:...	67871	1.626e+05	99%	0.0	99.11%	5104557	CP007592.1
☑	Salmonella enterica subsp. enterica serovar Indiana strain S530 chromosome, complete genome	Salmonella enterica s...	66330	2.093e+05	98%	0.0	99.55%	5059260	CP061118.1
☑	Salmonella enterica subsp. enterica serovar Indiana strain XZ14C1328 chromosome, complete genome	Salmonella enterica s...	66297	1.807e+05	100%	0.0	99.55%	4821996	CP102827.1
☑	Escherichia coli strain ABW_A19 chromosome, complete genome	Escherichia coli	57937	1.620e+05	94%	0.0	99.00%	5250599	CP067307.1
☑	Escherichia coli strain EC6563 chromosome, complete genome	Escherichia coli	57372	97420	91%	0.0	98.98%	4850951	CP095856.1
☑	Klebsiella pneumoniae strain 2019036D chromosome, complete genome	Klebsiella pneumoniae	57300	95088	96%	0.0	98.97%	5369757	CP047336.1
☑	Salmonella enterica subsp. enterica serovar Indiana strain SI174 chromosome, complete genome	Salmonella enterica s...	45426	1.400e+05	74%	0.0	99.38%	4929169	CP050756.1
☑	Salmonella enterica subsp. enterica serovar Indiana strain FJC33 chromosome, complete genome	Salmonella enterica s...	45421	1.484e+05	67%	0.0	99.37%	4941329	CP041699.1
☑	Escherichia coli strain SM107 chromosome, complete genome	Escherichia coli	39803	1.724e+05	99%	0.0	99.95%	5166015	CP130667.1
☑	Salmonella sp. SJTUF14152 chromosome, complete genome	Salmonella sp. SJTUF...	37986	1.926e+05	98%	0.0	99.74%	4995432	CP064671.1
☑	Escherichia coli strain ECJXMCRH7 chromosome, complete genome	Escherichia coli	32913	81125	65%	0.0	98.34%	4864517	CP067342.1
☑	Escherichia coli strain EC12 chromosome, complete genome	Escherichia coli	32904	1.333e+05	72%	0.0	98.32%	4982993	CP060933.1
☑	Escherichia coli strain W444 chromosome, complete genome	Escherichia coli	32897	1.191e+05	65%	0.0	98.32%	4747817	CP122507.1
☑	Escherichia coli strain AHS8C65RI chromosome, complete genome	Escherichia coli	32891	91039	65%	0.0	98.32%	5080150	CP125885.1
☑	Klebsiella pneumoniae strain YZ22CK024 chromosome, complete genome	Klebsiella pneumoniae	32830	67335	64%	0.0	98.37%	5304021	CP110145.1
☑	Klebsiella pneumoniae strain LSH-KPN25 chromosome	Klebsiella pneumoniae	32814	73252	66%	0.0	98.36%	5441147	CP040179.1
☑	Klebsiella pneumoniae strain LSH-KPN25 chromosome, complete genome	Klebsiella pneumoniae	32814	73252	66%	0.0	98.36%	5441138	CP040391.1
☑	Klebsiella pneumoniae strain EAN23 chromosome, complete genome	Klebsiella pneumoniae	32814	85883	66%	0.0	98.36%	5452022	CP039957.1
☑	Klebsiella pneumoniae strain 19KM57 chromosome, complete genome	Klebsiella pneumoniae	32814	85883	66%	0.0	98.36%	5452451	CP039948.1
☑	Klebsiella pneumoniae strain 19KM28 chromosome, complete genome	Klebsiella pneumoniae	32814	85883	66%	0.0	98.36%	5453228	CP039945.1
☑	Klebsiella pneumoniae strain STLE2 chromosome, complete genome	Klebsiella pneumoniae	32808	1.001e+05	76%	0.0	98.35%	5457824	CP058857.1
☑	Klebsiella pneumoniae strain SWHIN_114 chromosome, complete genome	Klebsiella pneumoniae	32808	1.001e+05	76%	0.0	98.35%	5459081	CP055087.1
☑	Klebsiella pneumoniae strain 19KM267 chromosome, complete genome	Klebsiella pneumoniae	32808	85866	66%	0.0	98.35%	5452021	CP049947.1
☑	Klebsiella pneumoniae strain EAN40 chromosome, complete genome	Klebsiella pneumoniae	32808	85866	66%	0.0	98.35%	5452088	CP039960.1
☑	Klebsiella pneumoniae strain 18KM2813 chromosome, complete genome	Klebsiella pneumoniae	32808	85866	66%	0.0	98.35%	5452451	CP039942.1

Supplementary Figure S1. Blastn results of the genomic island analysis based on the NCBI database. Blastn results of the genomic island analysis showed that other Enterobacteriaceae also have drug-resistant genomic islands similar to that found in this study.

Supplementary Table S1. Potential genomic islands of *S.* Indiana S1467

Island start	Island end	Length	Method	Note
811,436	815,615	4,179	IslandPick	
1,280,753	1,286,172	5,419	IslandPick	
1,895,017	1,923,701	28,684	IslandPick	
2,278,029	2,297,338	19,309	IslandPick	
2,297,964	2,304,743	6,779	IslandPick	
2,305,063	2,309,539	4,476	IslandPick	
2,990,700	2,996,893	6,193	IslandPick	
3,004,045	3,008,581	4,536	IslandPick	
3,020,555	3,025,277	4,722	IslandPick	
3,728,009	3,732,239	4,230	IslandPick	
3,749,721	3,755,132	5,411	IslandPick	
4,269,326	4,274,310	4,984	IslandPick	
4,274,592	4,289,123	14,531	IslandPick	
4,472,550	4,486,656	14,106	IslandPick	
4,493,911	4,501,405	7,494	IslandPick	
26,419	31,647	5,228	SIGI-HMM	
59,103	65,539	6,436	SIGI-HMM	
127,350	135,702	8,352	SIGI-HMM	
458,433	465,086	6,653	SIGI-HMM	
649,677	653,879	4,202	SIGI-HMM	
662,147	666,300	4,153	SIGI-HMM	
667,932	676,942	9,010	SIGI-HMM	
791,312	799,071	7,759	SIGI-HMM	
821,644	833,455	11,811	SIGI-HMM	
837,088	841,641	4,553	SIGI-HMM	
847,357	864,245	16,888	SIGI-HMM	
1,036,028	1,040,993	4,965	SIGI-HMM	
1,097,670	1,105,126	7,456	SIGI-HMM	
1,236,675	1,244,287	7,612	SIGI-HMM	
1,565,810	1,573,520	7,710	SIGI-HMM	
1,580,485	1,599,572	19,087	SIGI-HMM	

1,744,359	1,749,587	5,228	SIGI-HMM	
1,754,055	1,760,056	6,001	SIGI-HMM	
1,862,799	1,871,593	8,794	SIGI-HMM	
1,900,613	1,906,805	6,192	SIGI-HMM	
1,912,869	1,923,631	10,762	SIGI-HMM	
1,965,962	1,975,930	9,968	SIGI-HMM	
2,059,310	2,080,531	21,221	SIGI-HMM	
2,194,254	2,199,381	5,127	SIGI-HMM	
2,200,804	2,205,609	4,805	SIGI-HMM	
2,294,521	2,304,421	9,900	SIGI-HMM	
2,447,790	2,453,946	6,156	SIGI-HMM	
2,472,993	2,480,114	7,121	SIGI-HMM	
2,699,759	2,704,253	4,494	SIGI-HMM	
2,707,577	2,725,080	17,503	SIGI-HMM	
2,867,848	2,880,494	12,646	SIGI-HMM	
3,012,059	3,025,095	13,036	SIGI-HMM	
3,027,925	3,039,827	11,902	SIGI-HMM	
3,324,620	3,329,815	5,195	SIGI-HMM	
3,507,174	3,513,331	6,157	SIGI-HMM	
3,736,119	3,742,462	6,343	SIGI-HMM	
3,749,132	3,763,389	14,257	SIGI-HMM	
3,771,041	3,775,319	4,278	SIGI-HMM	
3,779,684	3,783,859	4,175	SIGI-HMM	
3,784,591	3,791,052	6,461	SIGI-HMM	
3,809,756	3,814,567	4,811	SIGI-HMM	
3,818,024	3,822,552	4,528	SIGI-HMM	
4,268,595	4,277,653	9,058	SIGI-HMM	
4,459,881	4,465,199	5,318	SIGI-HMM	
4,467,069	4,474,820	7,751	SIGI-HMM	
4,476,629	4,485,029	8,400	SIGI-HMM	
4,488,730	4,498,593	9,863	SIGI-HMM	
4,506,449	4,514,615	8,166	SIGI-HMM	
4,594,088	4,598,688	4,600	SIGI-HMM	
646,462	697,754	51,292	IslandPath-DIMOB	This study

789,835	871,216	81,381	IslandPath-DIMOB
1,234,423	1,244,287	9,864	IslandPath-DIMOB
1,286,852	1,297,162	10,310	IslandPath-DIMOB
1,888,011	1,925,369	37,358	IslandPath-DIMOB
2,052,842	2,080,531	27,689	IslandPath-DIMOB
2,285,046	2,309,408	24,362	IslandPath-DIMOB
2,866,756	2,880,494	13,738	IslandPath-DIMOB
2,981,118	3,038,641	57,523	IslandPath-DIMOB
3,738,065	3,791,052	52,987	IslandPath-DIMOB
3,810,494	3,842,754	32,260	IslandPath-DIMOB
4,255,783	4,280,932	25,149	IslandPath-DIMOB
4,450,968	4,514,615	63,647	IslandPath-DIMOB
4,571,228	4,596,681	25,453	IslandPath-DIMOB
